# Case report: A double pathogenic mutation in a patient with late-onset MELAS/PEO overlap syndrome

**DOI:** 10.3389/fneur.2022.927823

**Published:** 2022-08-11

**Authors:** Qiu Yan Zhao, Wen Zhao Zhang, Xue Lian Zhu, Fei Qiao, Li Yuan Jia, Bi Li, Yong Xiao, Han Chen, Yu Zhang, Yun Guo Chen, Yong Liang Wang

**Affiliations:** ^1^Department of Neurology, The Fourth Division Hospital of Xinjiang Production and Construction Corps, Yining, China; ^2^Department of Clinical Laboratory, Zhenjiang Hospital of Chinese Traditional and Western Medicine, Zhenjiang, China; ^3^Department of Orthopedics, The Fourth Division Hospital of Xinjiang Production and Construction Corps, Yining, China; ^4^Department of Medical Imaging, The Fourth Division Hospital of Xinjiang Production and Construction Corps, Yining, China; ^5^Department of Cardiovascular, The Fourth Division Hospital of Xinjiang Production and Construction Corps, Yining, China; ^6^Department of Interventional, The Fourth Division Hospital of Xinjiang Production and Construction Corps, Yining, China

**Keywords:** MELAS, PEO, mutation, Chinese, gene

## Abstract

Mitochondrial encephalopathy, lactic acidosis, and stroke-like episodes (MELAS) and progressive external ophthalmoplegia (PEO) are established phenotypes of mitochondrial disorders. They are maternally-inherited, multisystem disorder that is characterized by variable clinical, biochemical, and imaging features. We described the clinical and genetic features of a Chinese patient with late-onset MELAS/PEO overlap syndrome, which has rarely been reported. The patient was a 48-year-old woman who presented with recurrent ischemic strokes associated with characteristic brain imaging and bilateral ptosis. We assessed her clinical characteristics and performed mutation analyses. The main manifestations of the patient were stroke-like episodes and seizures. A laboratory examination revealed an increased level of plasma lactic acid and a brain MRI showed multiple lesions in the cortex. A muscle biopsy demonstrated ragged red fibers. Genetic analysis from a muscle sample identified two mutations: TL1 m.3243A>G and POLG c.3560C>T, with mutation loads of 83 and 43%, respectively. This suggested that mitochondrial disorders are associated with various clinical presentations and an overlap between the syndromes and whole exome sequencing is important, as patients may carry multiple mutations.

## Introduction

Mitochondrial disorders are a heterogeneous group of disorders that may affect multiple organ systems, displaying marked phenotypic and genetic heterogeneity. Mitochondrial encephalopathy, lactic acidosis, and stroke-like episodes (MELAS) is a rare disorder with multi-system involvement. Stroke-like episodes are the core symptom of MELAS, which is manifested as sudden hemiplegia, aphasia, psychiatric symptoms, and hemianopia/cortical blindness. Characteristic brain MRI shows large, long T2 signals at the junction of parietal, occipital, and temporal lobes (1). Most MELAS patients develop stroke-like symptoms before the age of 40, whilst only 1 to 6% of patients develop symptoms after this age, which is called late-onset MELAS (2). Other manifestations of MELAS include epilepsy, headache, cognitive decline, exercise intolerance, diabetes, deafness, stature short, and hairiness. Muscle biopsies have revealed characteristic ragged red fibers (RRF). More than 80% of patients have TL1 m.3243A>G mutation in their mitochondrial DNA (mtDNA) (3). Progressive external ophthalmoplegia (PEO) often overlaps with other variable symptoms. The characteristic clinical presentations of PEO are ptosis, external ophthalmoplegia, muscle weakness, high-frequency sensorineural hearing loss, and dysphagia (4). POLG is the most common gene causing PEO and is associated with complex and severe phenotypes. Besides, a variety of genes have been implicated in the development of PEO (4). Here, we summarize the clinical, pathological, and gene mutation characteristics of a double pathogenic mutation in a Chinese patient.

## Case presentation

**Figure 1:**
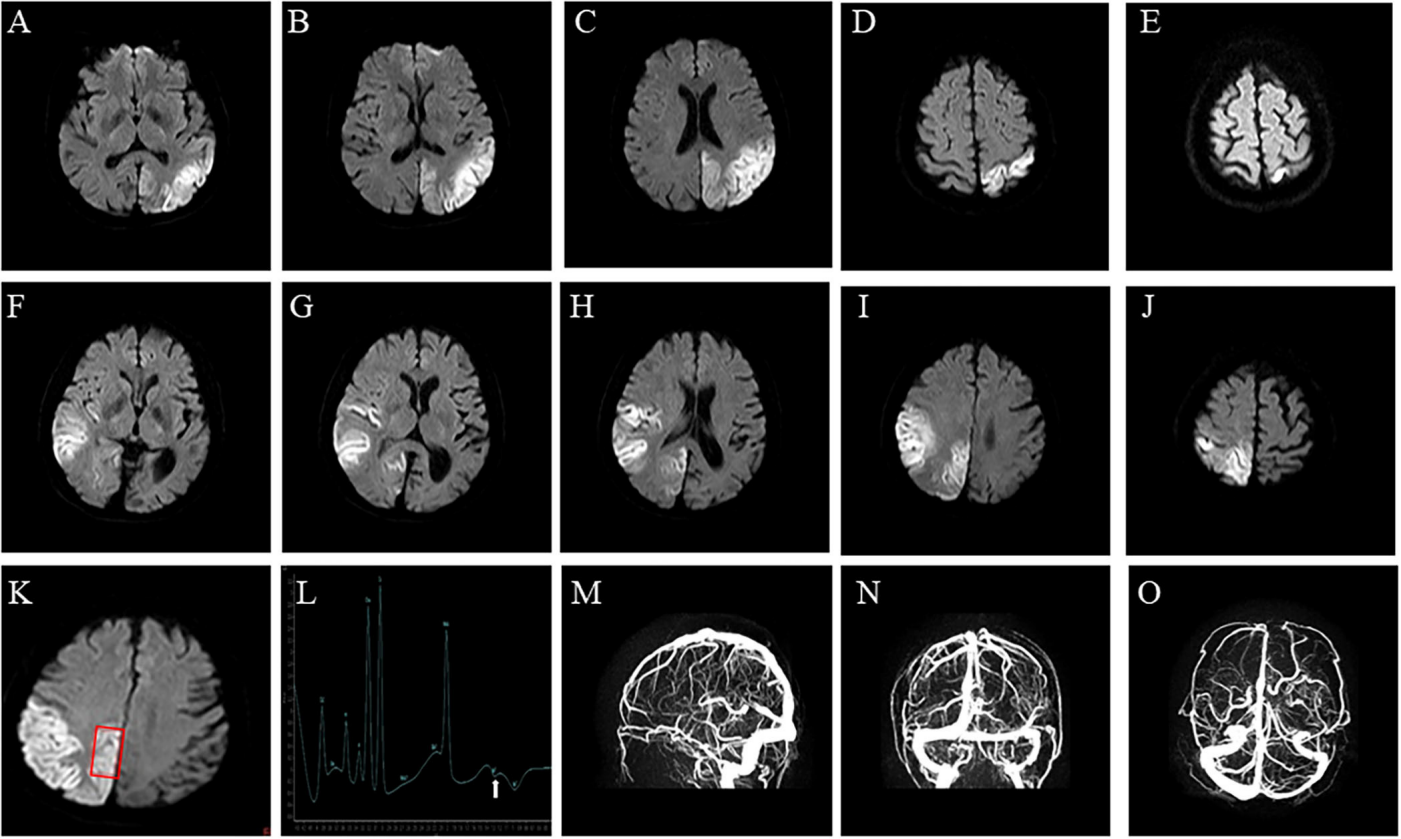
Brain imaging. **(A**–**E)** Axial diffusion-weighted of brain MRI on Diffusion-Weighted Imaging shows water restriction in the left occipital lobes, related to the first stroke-like episode, and **(F**–**J)** in the right temporal occipital lobe, related to the second stroke-like episode. **(K,L)** Brain Magnetic Resonance Spectroscopy shows abnormal lactate metabolism, at 1.3 ppm. **(M–O)** Brain Magnetic Resonance Venography shows no abnormality in the dural sinuses.

A 48-year-old right-handed woman was admitted to our hospital (August 2021) with complaints of right hemiparesis and cognitive impairment without ptosis and dysarthric. She had experienced recurrent stroke-like episodes. A brain MRI showed a lesion in the left occipital lobe (Figures 1A–E) and she was diagnosed with ischemic stroke but recovered without sequelae. MMSE and MOCA could not be completed at the time of the patient's onset, and the patient's MMSE score was 30 points and MOCA score was 30 points before discharge. She was discharged with outpatient follow-up in the stroke clinic.

Then, 4 months after her discharge, she was admitted to our hospital due to the acute onset of disorientation, and behavioral and speech disturbances. She denied any history of migraine-type headaches, seizures, dementia, muscle weakness, vomiting, hearing loss, and endocrinopathies. In her past medical history, her birth, childhood development, and early adulthood were normal. No neurological symptoms were observed in the patient's maternal relatives.

On examination, the patient was thin (45 kg) and of short stature (155 cm). The patient's vital signs were within the normal range. She was afebrile and auscultation revealed normal heart sounds and bilateral, equal air entry. Her abdomen was soft and non-tender and bowel sounds were present. During the neurological examination, the patient's cognitive function, such as numeracy, timing, and orientation, became impaired. She demonstrated bilateral ptosis and her speech was dysarthric (Supplementary Figure 1). Tone and power were normal in all muscle groups. Romberg's test was positive. Her gait was broad-based and ataxic. Nerve conduction studies showed primarily sensory peripheral neuropathy. There was no Babinski sign.

The patient's plasma lactate level was 2.8 mmol/L (normal range.5-2.2 mmol/L), whilst other measurements, such as complete blood count, glycemia, creatinine phosphokinase, liver and kidney function tests, electrolytes and thyroid function tests, were all normal. A cerebrospinal fluid analysis showed a normal cell count, protein, and glucose levels. An echocardiograph, electromyograph, and chest CT scan were also normal. However, a brain MRI revealed a large diffusion weighted water restriction lesion in the right temporo-occipital lobe (Figures 1F–J). No arterial occlusion was observed. A 1H-magnetic resonance spectroscopy (MRS) revealed abnormal lactate concentrations in the parietal lobe lesion (Figures 1K,L). A brain magnetic resonance venography (MRV) was performed to exclude cerebral vein thrombosis. The results of this were normal (Figures 1M–O).

On the fourth day after admission, the patient had a brief (30 s) right-side tonic head deviation during which she remained unresponsive to verbal and painful stimuli. There was no tongue biting or loss of urinary or bowel control. An EEG showed the left posterior temporo-occipital region (T5 and O1), with sharp and slow-wave discharges that spread to the contralateral occipital region, in a background of diffuse slow wave activity. Levetiracetam (1 g daily in divided doses) was prescribed. The level of cognitive function had improved by the 10th day after admission and the patient was discharged on day 14 day, with Levetiracetam, L-carnitine, coenzyme-Q 10, and multivitamins. After 2 months of follow-up, the patient exhibited slow actions, but was well-oriented and had no residual behavioral disturbance. No deficit was noted in memory, language, visuospatial, or executive functions. No further seizures had occurred and the patient was autonomous in daily activities such as walking, dressing, and eating. The patient did not report any side effects of the treatment. Levetiracetam, L-Arginine, and Coenzime Q are prescribed for long-term use.

**Figure 2:**
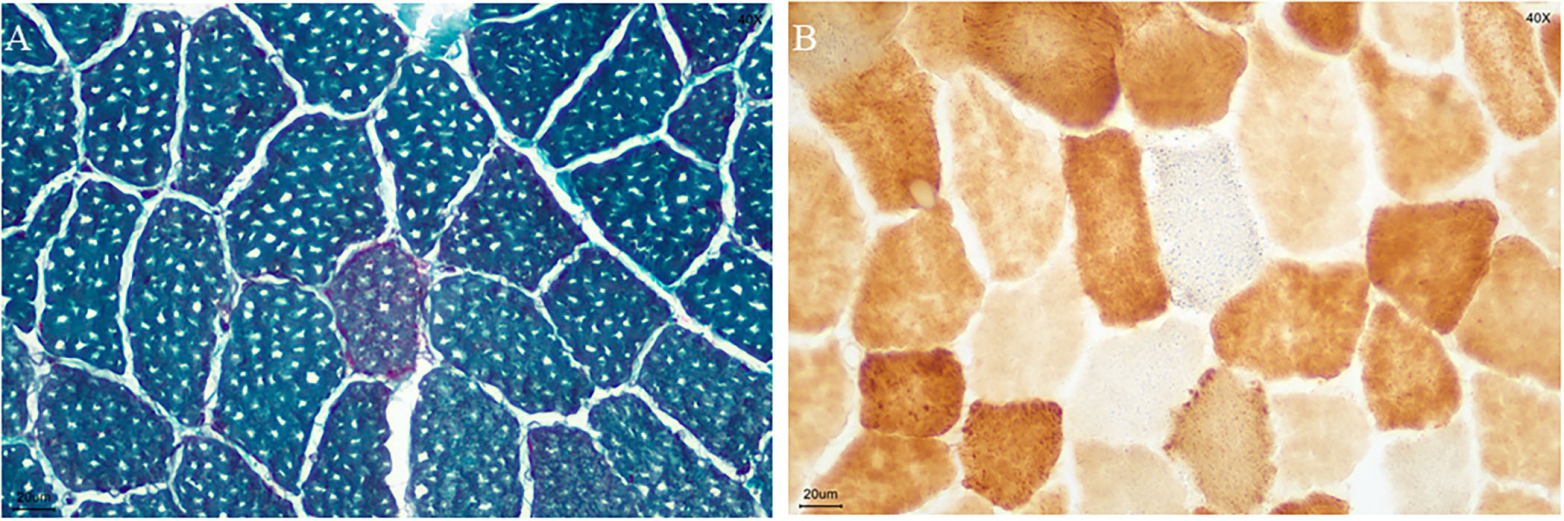
Biopsy specimen. **(A)** ragged red fibers (MGT staining**)**; **(B)** COX-negative and SDH-positive fibers in blue (COX/SDH double staining) (bar = 20μm).

A biopsy was performed on the quadriceps muscle. The tissue was frozen and then cut into 7 mm sections, which were stained in accordance with standard histological and enzymatic histochemical procedures. The staining included the use of H&E, modified Gomori trichrome (MGT), periodic acidic Schiff (PAS), oil red O (ORO), nicotinamide adenine dinucleotide tetrazolium reductase (NADH-TR), succinate dehydrogenases (SDH), cytochrome C oxidase (COX), COX/SDH double staining, and non-specific esterase (NSE). The analysis showed ragged red fibers (Figure 2A), and SDH-positive fibers and gave a COX-negative result (Figure 2B).

**Figure 3:**
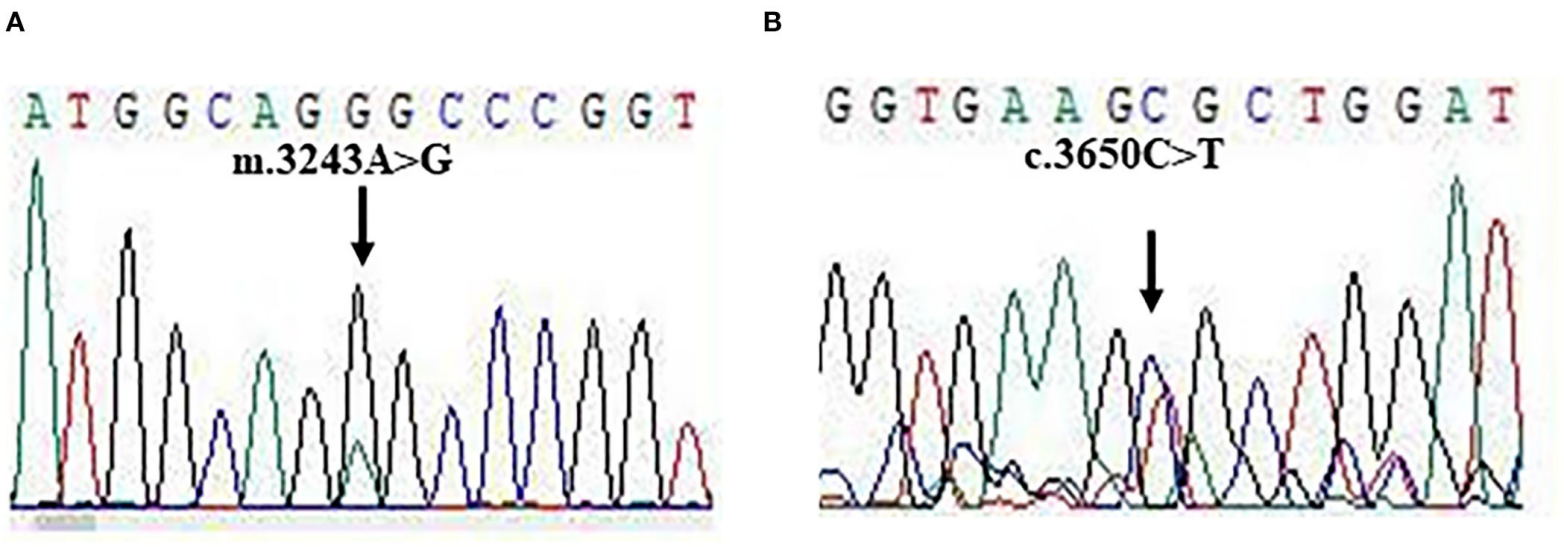
The gene mutations. **(A,B)** Sanger sequencing for m.3243A>G and c.3560C>T mutations.

For molecular diagnosis, total genomic DNA was isolated from blood and muscle sample, respectively. Whole exome sequencing (WES) was performed as described in previous studies. Mitochondrial disorders associated with pathogenic mutations were found in DNA from muscle samples, but not from blood samples. Two pathogenic mutations: TL1 m.3243A>G and POLG c.3560C>T were identified in our patient (Figures 3A,B). The mutations were heteroplasmic, with mutant mtDNA/total mtDNA ratios of 83 and 43%, respectively. The results of the WES were confirmed by Sanger sequencing.

## Discussion

Mitochondrial encephalopathy, lactic acidosis, and stroke-like episodes syndrome were first described as a unique mitochondrial disorder in 1984 (5), with inheritance through the maternal lineage. Although it usually occurs before the age of 40, cases of adult-onset MELAS have been reported (2, 3). Clinical symptoms, such as homonymous hemianopsia, seizures, migraine headaches, stroke-like episodes, and an altered mental status, are attributed to an impaired energy supply in high energy demanding organs such as the brain and muscle. Stroke-like episodes are the most common clinical features (1–3) and MELAS should be considered in adult patients with stroke-like episodes, without cerebrovascular risk factors. These episodes may show a variety of neurological symptoms, such as homonymous hemianopsia, altered mental status, and seizures. In MELAS, most strokes happen in the occipital lobe but tend not to have a vascular distribution. The corresponding changes observed during brain imaging are very helpful for the diagnosis of MELAS syndrome. In addition, most muscle biopsies show RRF (1–3). The mutation hotspots observed in MELAS syndrome are m.3243A>G, m.3271T>C and m.13513G>A (5). The pathogenic mechanism of MELAS has not been completely elucidated, but the main hypotheses propose that these mutations interfere with protein synthesis in the mitochondria, which results in a deficiency of respiratory chain enzyme complexes I and IV and the reduction of adenosine triphosphate (ATP).

Progressive external ophthalmoplegia mainly presents with bilateral ptosis and ophthalmoparesis progressing in severity over time. POLG is the most common gene associated with PEO, both in autosomal dominant or recessive form. POLG is located on chromosome 15q25 with 23 exons. POLG mutation is associated with at least five major phenotypes, including Alpers-Huttenlocher syndrome (AHS), PEO with or without sensory ataxic neuropathy, dysarthria, and ophthalmoplegia (SANDO), childhood myocerebrohepatopathy spectrum (MCHS), myoclonic epilepsy myopathy sensory ataxia (MEMSA), and the ataxia neuropathy spectrum (ANS). Patients with POLG-associated encephalopathy have a distinct phenotype and neuroimaging characterized by predominant posterior ischemic lesions (4).

Our patient was diagnosed with MELAS/PEO overlap syndrome by the typical clinical symptoms, the results of a muscle biopsy, and the identification of the double pathogenic mutations. MELAS patients have a high prevalence of epilepsy (1–5) and our patient had focal paroxysmal activity in the stroke-like lesion. The symptoms of our patient improved significantly while on treatment and when oral Levetiracetam, L-arginine, and coenzyme-Q 10 were discontinued. If epilepsy is involved, pharmacological antiepileptic treatment should be routinely prescribed to patients.

In summary, we reported a patient with late-onset MELAS/PEO to overlap syndrome who harbored the TL1 m.3243A>G and POLG c.3560C>T. Mitochondrial disorders are associated with various clinical presentations and an overlap between the syndromes and WES is important for the diagnosis and genetic counseling of mitochondrial disorders patients other than the screening of mutation hotspots. Oral L-arginine and coenzyme-Q 10 were effective for this MELAS patient but their effectiveness should be validated by multicenter, randomized, controlled trials to demonstrate safety and efficacy.

## Data availability statement

The datasets presented in this article are not readily available because of ethical and privacy restrictions. Requests to access the datasets should be directed to the corresponding authors.

## Ethics statement

Written informed consent was obtained from the individual(s) for the publication of any potentially identifiable images or data included in this article.

## Author contributions

YZ, YC, and YW collected patient clinical data and were major contributors to writing the manuscript. QZ, XZ, and WZ were in charge of analyzing and interpreting the patient data and revising the draft critically for important intellectual content. FQ, LJ, BL, YX, and HC were responsible for collecting the clinical data. All authors read and approved the final manuscript.

## Funding

This study was supported by the Project of Shanghai Science and Technology Commission (22015831100).

## Conflict of interest

The authors declare that the research was conducted in the absence of any commercial or financial relationships that could be construed as a potential conflict of interest.

## Publisher's note

All claims expressed in this article are solely those of the authors and do not necessarily represent those of their affiliated organizations, or those of the publisher, the editors and the reviewers. Any product that may be evaluated in this article, or claim that may be made by its manufacturer, is not guaranteed or endorsed by the publisher.
