# Supplementary figures and images for: Case report: A double pathogenic mutation in a patient with late-onset MELAS/PEO overlap syndrome
Source: Front Neurol. 2022 Aug 11;13:927823. doi: 10.3389/fneur.2022.927823 (PMC9414032; doi:10.3389/fneur.2022.927823)

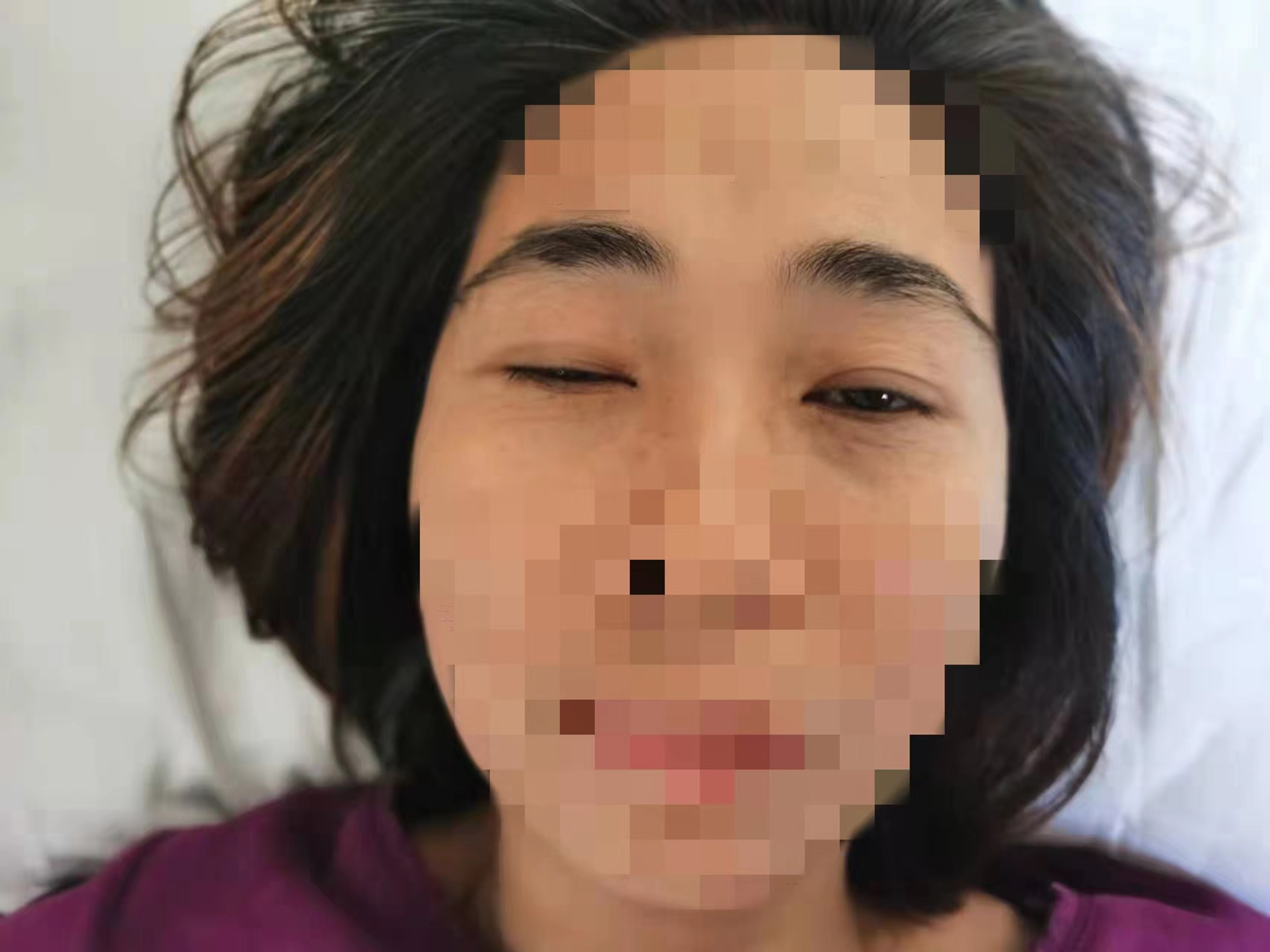

Supplement: Supplementary file 1 [file Image_1.JPEG]
